# Bioactive IGF-1 release from collagen–GAG scaffold to enhance cartilage repair in vitro

**DOI:** 10.1007/s10856-014-5325-y

**Published:** 2015-01-11

**Authors:** Leanne M. Mullen, Serena M. Best, Siddhartha Ghose, John Wardale, Neil Rushton, Ruth E. Cameron

**Affiliations:** 1 Department of Materials Science and Metallurgy, University of Cambridge, Pembroke Street, Cambridge, CB2 3QZ UK; 2 Tigenix Limited, Byron House, Cambridge Business Park, Milton Road, Cambridge, UK; 3 Orthopaedic Research Unit, University of Cambridge, Cambridge, UK

## Abstract

Tissue engineering is a promising technique for cartilage repair. Toward this goal, a porous collagen–glycosaminoglycan (CG) scaffold was loaded with different concentrations of insulin-like growth factor-1 (IGF-1) and evaluated as a growth factor delivery device. The biological response was assessed by monitoring the amount of type II collagen and proteoglycan synthesised by the chondrocytes seeded within the scaffolds. IGF-1 release was dependent on the IGF-1 loading concentration used to adsorb IGF-1 onto the CG scaffolds and the amount of IGF-1 released into the media was highest at day 4. This initial IGF-1 release could be modelled using linear regression analysis. Osteoarthritic (OA) chondrocytes seeded within scaffolds containing adsorbed IGF-1 deposited decorin and type II collagen in a dose dependent manner and the highest type II collagen deposition was achieved via loading the scaffold with 50 μg/ml IGF-1. Cells seeded within the IGF-1 loaded scaffolds also deposited more extracellular matrix than the no growth factor control group thus the IGF-1 released from the scaffold remained bioactive and exerted an anabolic effect on OA chondrocytes. The effectiveness of adsorbing IGF-1 onto the scaffold may be due to protection of the molecule from proteolytic digestion allowing a more sustained release of IGF-1 over time compared to adding multiple doses of exogenous growth factor. Incorporating IGF-1 into the CG scaffold provided an initial therapeutic burst release of IGF-1 which is beneficial in initiating ECM deposition and repair in this in vitro model and shows potential for developing this delivery device in vivo.

## Introduction

The unique biomechanical properties of articular cartilage are attributed to the complex zonal arrangement of its constituent macromolecules, collagen and proteoglycan. Cells known as chondrocytes synthesise and maintain the extracellular matrix (ECM) which enables movement under almost frictionless and wear resistant conditions [4]. Articular cartilage defects are common and present a major risk factor in the development of osteoarthritis (OA) in later life, yet articular cartilage has a limited capacity for self-repair. Moreover, surgical treatment of cartilage defects mainly yields suboptimal fibrous repair tissue. Inadequate cartilage repair tissue fails to withstand the biomechanical forces acting on the joint and degrades over time hence much research has focused on utilising tissue engineering strategies to yield a fully functional, long lasting repair tissue [46]. Tissue engineering is the persuasion of the body to heal itself through the delivery of cells, biomolecules and scaffolds [55]. Towards this goal, this research describes loading a collagen–glycosaminoglycan (CG) scaffold with bioactive molecules and cells to enhance articular cartilage regeneration within an in vitro model.

Scaffold design has centred on developing ECM matrix analogues. Scaffold microstructure significantly affects cell adhesion, migration and proliferation within the matrix [8]. For cartilage tissue engineering, highly porous scaffolds with large surface areas and adequate mechanical strength are desirable, while a pore size of 100–500 μm has been reported to be optimal for this repair [8, 15, 22, 23, 41]. The CG scaffold used in this study is composed of type I collagen and chondroitin sulphate which is chemically cross-linked to enhance its mechanical properties and freeze-dried to create a porous microstructure. The CG scaffolds possess highly interconnected porous architectures with an average pore size of 216 ± 39 µm [38]. CG scaffolds are promising regeneration templates for many different tissues including; tendon, meniscal tissue, conjunctiva, heart valves, tendon and ligaments [3, 6, 18, 20, 35, 44]. The most extensive research has focused on skin and peripheral nerve regeneration and has yielded substantial success, as demonstrated by FDA approval [56]. Collagen is a key component of many biomedical devices, while the chondroitin sulphate within the scaffold provides compressive resistance and a microenvironment for cells that is similar to native tissue. In vivo, cell–ECM interactions provide adequate signals to cells to induce or maintain a desired state of cell differentiation. Growth factors are soluble proteins which stimulate cell proliferation and differentiation and can be used to aid cell migration, and to increase matrix production [18, 23, 47]. Thus these proteins play an important role in in vitro tissue engineering [53] and may need to be added exogenously to achieve optimal tissue repair.

Mature chondrocytes exhibit high metabolic activity and characteristically synthesise type II collagen and large aggregating proteoglycans such as aggrecan [4]. The ability of chondrocytes to maintain the metabolic homeostasis of the matrix decreases with ages [5, 7, 32]. Characteristics of these aged chondrocytes include; the synthesis of smaller, less regular aggrecan, reduced proteoglycan synthesis, telomere shortening and a diminished response to insulin-like growth factor (IGF-1) [31, 33]. Osteoarthritic chondrocytes possess a significantly lower proliferative activity than normal chondrocytes [1, 21, 30] and poor responsiveness to growth factors [37]. However, human articular chondrocytes can be isolated from mature articular cartilage via enzymatic digestion and expanded in vitro. Their expression of type II collagen and proteoglycan demonstrates that they partially maintain their phenotype [25] and a study of a micromass pellet culture of human OA chondrocyte indicated that these chondrocytes are capable of differentiation and proliferation in vitro comparable with normal chondrocytes for 14 days [49]. Furthermore, in human cartilage specimens with advanced OA some chondrocytes continue to express link protein and type II collagen mRNA, indicating that they have remained anabolically active [2]. Therefore it was of interest to us into establish whether IGF-1 would enhance the OA chondrocytes phenotype when delivered via a biomimetic scaffold.

IGF-1, an anabolic growth factor involved in cartilage development and homeostasis [12, 51] has been extensively investigated for use in articular cartilage repair [10, 12, 17, 34, 40, 51]. Fortier et al. [12] demonstrated that the addition of 10–100 ng/ml IGF-1 enhanced the levels of proteoglycan and type II collagen synthesised by chondrocytes seeded in fibrin matrices and that the cells maintained their phenotype in vitro. In addition, IGF-1 also protects the ECM from interleukin-1 (IL-1) and tumour necrosis factor α-mediated degradation during cartilage injury [14, 16, 52]. In vivo, IGF-1 loaded fibrin matrices have been used to repair full thickness articular cartilage defects in horses [44]. These loaded matrices enhanced the formation of hyaline like repair tissue and had better adhesion to the subchondral bone compared to controls. Moreover, Tuncel et al. [51] reported that collagen sponges loaded with 5 µg IGF-1 enhanced the tissue response and produced significantly better quality neocartilage compared with the collagen sponge controls in a rabbit osteochondral defect model. Madry et al. [34] demonstrated that scaffolds filled with IGF-I-over-expressing chondrocytes yielded markedly improved osteochondral repair compared with control scaffolds.

IGF-1 plays a key role in articular cartilage maintenance during adult life; stimulating matrix protein production and inhibiting degradation and cell death [28, 29, 50]. The addition of exogenous IGF-1 to chondrocytes and articular cartilage explants blocks the IL-1 induced degradation of proteoglycans and aids chondrocyte survival [14, 24]. Low levels of IGF-1 are present in normal articular cartilage [36]. The effect of IGF-1 on articular cartilage is modulated during ageing and OA, resulting in reduced anabolism and an increase in matrix degradation. Schneiderman et al. [48] investigated the distribution of IGF-1 in normal and OA cartilage and found that in OA cartilage the concentration of all forms of IGF are higher and lie in the range necessary for the anabolic stimulation of chondrocytes. However, higher levels of IGF binding proteins (IGFBPs) have been found in OA chondrocytes and cartilage compared to those found in normal tissue and cells [11, 43]. This evidence suggests that the high levels of IGFBPs may be responsible for the hyporesponsiveness of OA chondrocytes to IGF-1.

IGF-1 was incorporated into the CG scaffold via sorption with aim to achieve the release of a therapeutic release of IGF-1 that would yield de novo hyaline articular cartilage tissue in vitro. This study aims to evaluate this scaffold as a growth factor delivery device and to assess the biological response in terms of the articular cartilage ECM proteins synthesised by the seeded chondrocytes. The binding and release of IGF-1 from the CG scaffold has been previously monitored using a radiolabeled method and the release profile was monitored over 14 days [9]. This study indicated that ionic interactions contribute to the binding and a burst release of IGF-1 occurred up to 24 h, followed by sustained release up to day 14. In the present study the amount of extracellular matrix proteins (type II collagen and proteoglycan) in the media or laid down on the scaffold were measured to assess the new ECM production within the model. CG scaffolds were also loaded using different IGF-1 loading concentrations (25, 50, 100 μg/ml IGF-1) to compare the biological effect on OA chondrocytes of the incorporation and release of different amounts of IGF-1 into the scaffold.

## Materials and methods

### Materials

#### CG scaffolds and cell culture

Type I insoluble collagen prepared from bovine skin (Devro Plc), and chondroitin-6-sulphate (Bioiberica) were used to prepare collagen–GAG scaffolds. 1-Ethyl-3-(3-dimethylaminopropyl) carbodiimide (EDC) and *N*-hydroxysuccinimide (NHS) were purchased from Sigma Aldrich. Human recombinant IGF-1 was purchased from R&D Systems. Human chondrocytes were derived from knee articular cartilage donated from patients (*N* = 3) undergoing total knee replacement surgery with full ethical consent 06/Q0108/213. Full depth articular cartilage was removed from femoral condyles and tibial plateaux using a scalpel. The tissue was then minced finely before incubating in a 0.2 % (w/v) solution of bacterial collagenase (Roche) in complete medium (DMEM, 10 % FCS plus antibiotics) overnight at 37 °C. Released cells were washed twice to remove any collagenase before plating on plastic. Chondrocytes were expanded and used at passage three in order to retain as much of the chondrogenic phenotype as possible. All experiments were carried out using cells derived from *N* = 3 individual donors.

#### Protein detection

Primary antibodies: collagen II: AVT6E3 (mouse monoclonal, kind gift from Anne Vaughan Thomas, University of Cardiff), decorin: (AF143, R&D Systems). Secondary antibodies (Sigma), anti-mouse peroxidase conjugated IgG (A0168, Sigma), anti-goat peroxidase conjugated IgG (A4174). Standards: Collagen II: human collagen II prepared by pepsinisation and salt fractionation from osteoarthritic knees from patients undergoing total joint replacement with full ethical consent, Recombinant decorin (Cat no. 143-DE, R&D systems). Immobilon PVDF (Millipore) membranes were used for western blotting. 1,9- Dimethyl-methylene blue (DMMB) was purchased from Sigma-Aldrich.

### Methods

#### Scaffold preparation

Collagen was added to an aqueous HCl solution (0.001 M) and left to swell overnight (17–20 h at 4 °C) to give a final concentration of 0.9 wt% collagen. The collagen slurry was blended for 1 h using a high energy blender (Turax (VWR Int.)) and cooling on ice. Chondroitin-6-sulphate (GAG) was dissolved in 0.001 M HCl, then added (0.08 wt%) and the slurry was blended for a further 30 min. The blended slurry was poured into glass moulds and freeze-dried (Virtis Advantage ES), using a constant cooling rate of 0.01 °C/min and a final freezing temperature of −12 °C. The discs were then chemically cross-linked using EDC/NHS/COOH at a molar ratio of 5:2:1 [6] and then freeze-dried using the same freeze-drying process. Final scaffold discs were 5 × 2 mm^2^ and weighed 1.5 ± 0.1 mg.

#### Cell culture

CG scaffolds were either incubated in media containing IGF-1 (25, 50 or 100 µg/ml) or media only for 24 h at 37 °C. The scaffolds were then rinsed and each scaffold was then seeded with 1 × 10^5^ primary OA chondrocytes which were allowed to attach to the scaffold for 2–3 h in media containing FCS (10 %) before continuing culture in 0.5 ml media for up to 14 days.

#### Measurement of released IGF-1

The IGF-1 released into the media from the scaffolds was measured using a Quantikine DG100 ELISA kit (R&D Systems) as described in the manufacturer’s instructions. Media samples from days 4, 7, 11, 14 were added to wells pre-coated with an antibody specific for IGF-1, washed (PBST) and incubated with peroxidase conjugated secondary antibody for 1 h. Further washing (PBST) was followed by the addition of the substrate and the reaction was stopped after 30 min by the addition of concentrated H_2_SO_4_. The absorbance was measured immediately at 544 nm in a Fluostar optima plate reader. The average amount of IGF-1 and standard error of the mean were used to report cumulative IGF-1 release. The table displays the average amount of IGF-1 released at each time point. The IGF-1 release up to day 4 was replotted against the loading concentration and linear regression analysis was performed, *P* < 0.05 was considered to be statistically significant.

#### DMMB assay

The total amount of proteoglycan synthesised by the cells was measured using the 1,9-dimethyl-methylene blue (DMMB) assay [7, 8]. Media samples (40 µl) from days 4, 7, 11, 14 of the experiment (*N* = 4) and CS standards (40 µl) were added to a 96 well plate and 250 µl of DMMB was added to each well. The absorbance was measured immediately at 544 nm in a Fluostar optima plate reader. The average amount of sulphated GAG and standard error of the mean were reported and data from each IGF-1 loading concentration was compared using a two tailed Student *t* test, *P* < 0.05 was considered to be statistically significant.

#### Western blotting

At each time point of the study scaffolds were removed and 300 µl of cell extraction buffer was added to each. These samples were rotated overnight and 100 µl of SDS sample buffer (4×) was added to each sample followed by heating at 60 °C for 30 min. Proteins were separated by SDS gel electrophoresis and Western blotted (*N* = 3 replicate samples from *N* = 3 donors) before probing for decorin and type II collagen. Specific bands for each protein were quantified using densitometry and standard curves of pure protein. The average amount of protein and standard error of the mean were reported and data from each IGF-1 loading concentration was compared using a two tailed Student *t*-test, *P* < 0.05 was considered to be statistically significant.

## Results

### IGF-1 released into the media

Scaffolds were loaded with different IGF-1 concentrations prior to cell-seeding and the media was collected on days 4, 7, 11 and 14 of the experiment. The amount of IGF-1 present in this media was measured using an ELISA assay. The release of IGF-1 for all loading groups was dominated by the release that occurs up to day 4, where 60–231 ng/ml IGF-1 was released compared with much lower release (1–13 ng/ml) for all later time points (Table 1).

**Table 1:** The amount of IGF-1 (ng/ml) released over 14 days from scaffolds loaded with each IGF-1 loading concentration

Time (Days)	IGF-1 release (ng/ml)					
	IGF-1 loading Concentration (µg/ml)					
	25	SEM	50	SEM	100	SEM
4	60.18	5.41	120.82	12.13	230.66	18.35
7	2.68	0.23	8.11	1.46	13.05	0.89
11	0.73	0.04	2.51	0.51	4.17	0.38
14	0.21	0.09	1.10	0.10	1.11	0.08

### Sulphated GAG release into the media

**Fig. 1:**
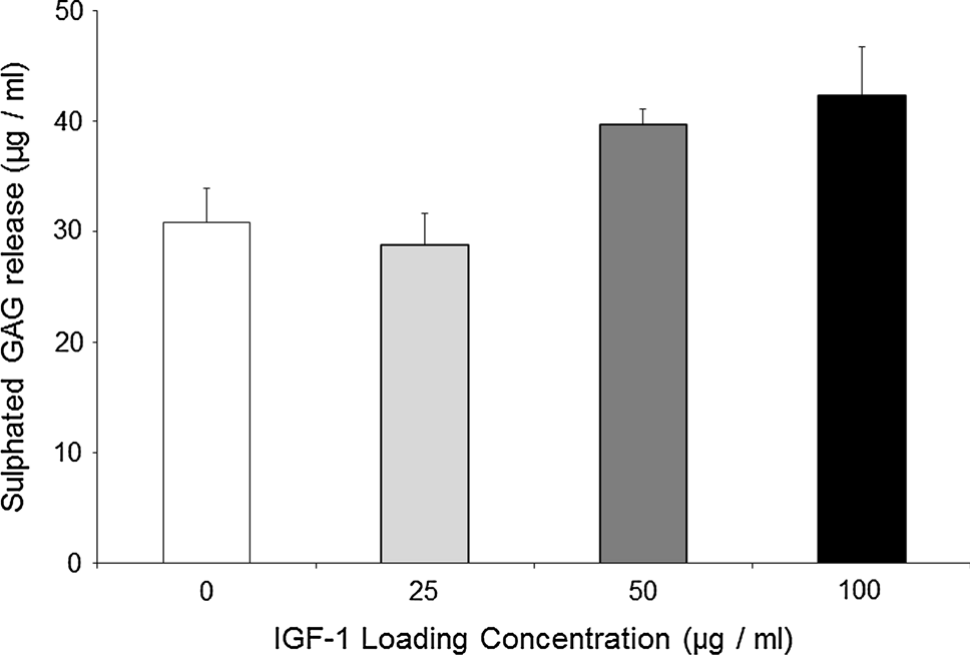
The amount of sulphated GAG released into the media (μg/ml) for each IGF-1 loading group at day 4. The significant differences (*P < 0.05) between groups, *N* = 4. The *error bars* represent the standard error of the mean

Chondrocytes grown in scaffolds loaded with 50 and 100 μg/ml IGF-1 produced a significantly higher amount of sulphated GAG in the media compared with the no growth factor control group after 4 days (Fig. 1).

### ECM deposition on the CG scaffold

#### Decorin

**Fig. 2:**
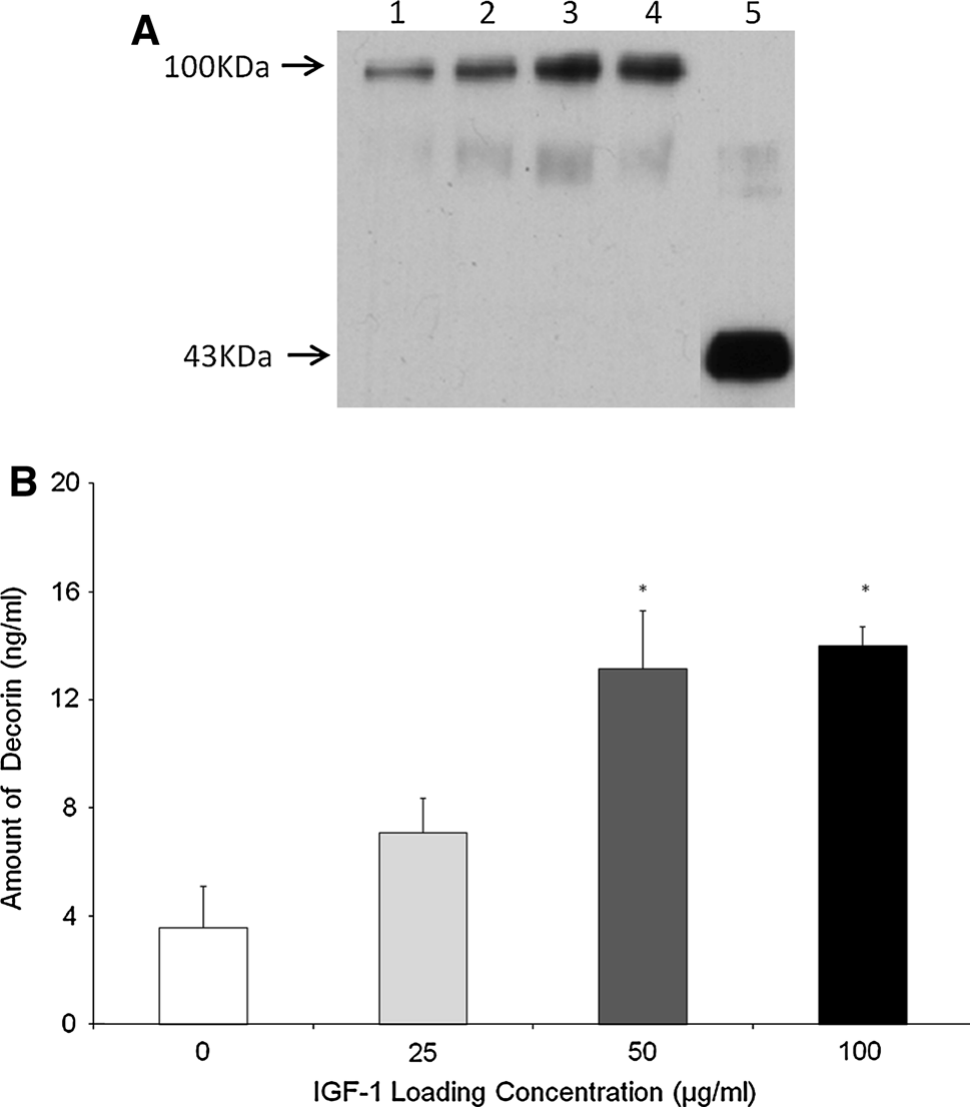
Decorin deposition on the Scaffold. **a** Western blot indicating decorin deposited by OA chondrocytes within the scaffold *1* 0 μg/ml IGF-1, *2* 25 μg/ml IGF-1, *3* 50 μg/ml IGF-1, *4* 100 μg/ml IGF-1, *5* Standard (recombinant decorin). **b** Quantification of western blot shown in Fig. 3a by densitometry. The significant differences (*P < 0.05) between groups. *N* = 4. The *error bars* represent the standard error of the mean

OA chondrocytes seeded with the scaffold demonstrated a dose dependent response of decorin deposition to increasing levels of IGF-1 (Fig. 2). This result was found to be statisically significant (0 vs. 50, 0 vs. 100, *P* < 0.05).

#### Type II collagen

**Fig. 3:**
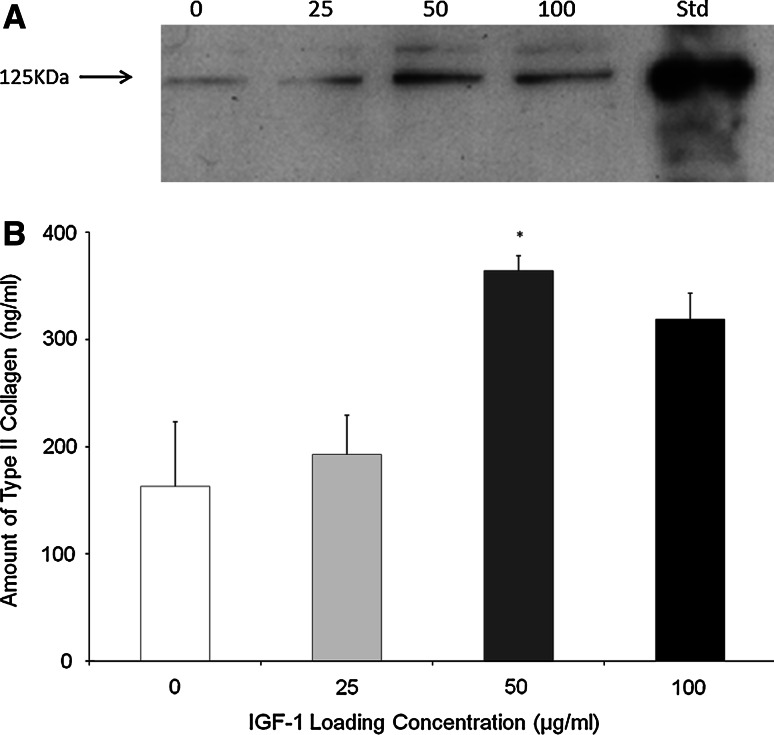
Type II Collagen Deposition on the Scaffold **a** Type II collagen Western Blot indicating the type II collagen deposited by OA chondrocytes within the scaffold *1* 0 µg/ml IGF-1, *2* 25 µg/ml IGF-1, *3* 50 µg/ml IGF-1, *4* 100 µg/ml IGF-1, *5* Standard (purified human type II collagen). **b** Quantification of western blot shown in (a) by densitometry. The significant differences (*P < 0.05) between groups. *N* = 3. The *error bars* represent the standard error of the mean

Similarly the levels of type II collagen deposited in the scaffold also responded positively to the IGF-1 that had been bound to the scaffold (Fig. 3) In this case the highest amount of type II collagen deposition was observed for scaffolds loaded with 50 μg/ml IGF-1 and this was significantly higher than the no growth factor control.

## Discussion

Previous studies have demonstrated that a steady or daily growth factor action would be advantageous [26, 39, 42, 50] and that 10 ng/ml IGF-1 is sufficient to stimulate the proliferative and metabolic activity of chondrocytes cultured in vitro while proteoglycan production is at a maximum using a dose of 100 ng/ml IGF-1 [12, 54]. The addition of IGF-1 to explants and monolayer cultures at concentrations of 10–200 ng/ml increased collagen type II DNA synthesis and inhibited proteoglycan degradation while 200 ng/ml also inhibited chondrocyte apoptosis in vitro [13, 27]. The benefit of adding different concentrations of endogenous IGF-1 (i.e. IGF-1 pre-loaded into the scaffold) on ECM synthesis was evaluated by comparing the amount of sulphated GAG and in the media, and the amounts of decorin and type II collagen deposited onto each CG scaffold. ECM was measured at day 4 as this corresponded with the highest release of IGF-1 from the scaffold into the media (Table 1).

Cells seeded within the endogenous IGF-1 group produced the highest level of sulphated GAG release, compared with the no growth factor control group (Fig. 1). However, this assay does not distinguish between newly synthesised sulphated GAG and the release of sulphated GAG from the CG scaffold. The known properties of IGF-1 would suggest that the increased release demonstrated in this study is probably due to growth factor mediated synthesis but in future, the incorporation of radiolabelled sulphate (^35^S) would be a better detection method as it would only detect newly synthesised sulphated GAG and thus would provide a more sensitive assay for comparing scaffold groups.

The OA chondrocytes from the IGF-1 groups yielded a significantly higher amount of decorin deposition onto the scaffold compared to the no growth factor control (*P* < 0.05) (Fig. 2). The results were similar to those obtained by the addition of 10–50 ng/ml exogenous IGF-1 (i.e. growth factor added to the medium) (data not shown). Therefore, one could speculate that the IGF-1 adsorbed onto scaffolds in the endogenous IGF-1 group may release at least 10–50 ng/ml IGF-1 within the first 4 days. The principal band detected on the decorin western blot is of a considerably higher molecular weight than purified recombinant decorin which runs at approximately 43 kDa. We achieve this pattern of bands on a routine basis whilst working with either whole cartilage or matrix deposited by chondrocytes in vitro. The recombinant standard used here is the unglycosylated core protein whereas the molecule that we extract from our cultures or tissue is partly or fully glycosylated with dermatan or chondroitin sulphate molecules leading to the increased molecular weight [45]. Decorin is also known to interact with a range of collagen molecules [18–20] and it is possible that some of these interactions also remain intact following our extraction procedures. We have used decorin in preference to aggrecan as a marker of proteoglycan synthesis to overcome the difficulties in western blotting aggrecan caused by its large molecular weight and lack of suitable antibodies.

Chondrocytes seeded within scaffolds pre-loaded with IGF-1 produced type II collagen deposition on the scaffold in a dose-dependent manner (Fig. 3). The effect of exogenous IGF-1 on type II collagen deposition was also compared with endogenous IGF-1 and the highest amount of type II collagen deposited onto the scaffold was found for cells within scaffolds containing endogenous IGF-1 (data not shown). The highest type II collagen deposition in this study was achieved via the addition of 50 μg/ml IGF-1 to the scaffold and deposition decreased as the IGF-1 concentration was increased to100 μg/ml. This trend may be due to the reported decrease in IGF-1 receptor (IGF-1R) mRNA levels induced by the addition of exogenous IGF-1 [9]. OA chondrocytes from older patients exhibit reduced anabolism, a reduced response to IGF stimulation and release higher levels of catabolic cytokines and IGFBPs. IGFBPs may potentate IGF binding to its receptor but high levels of these binding proteins facilitate the storage of IGF-1 and complexed IGF cannot bind to its receptor leading to hypo-responsiveness. Adding a high concentration of exogenous IGF-1 or IGF-1 bound to a scaffold may be necessary to saturate the high levels of IGFBPs released from these OA chondrocytes. Free IGF-1 has a short half life, but at high levels it can bind to both the IGFR1 and insulin receptor (IR) to exert an anabolic effect.

Adsorbing IGF-1 or other appropriate growth factors to a scaffold may partially protect the growth factor from degradation or inactivation allowing a longer anabolic effect. The effectiveness of adsorbing IGF-1 onto the scaffold may be due to protection of the molecule from proteolytic digestion allowing a more sustained release of IGF-1 over time compared to adding multiple doses of exogenous IGF-1. Hortensius and Harley also demonstrated a dose-dependent change in bioactivity to IGF-1 within CG scaffolds using an in vitro tenocyte model, this indicates that the growth factor remained bioactive within the matrix [19]. The stimulation of type II collagen production implied that these IGF-1 loaded scaffolds released a therapeutic dosage of IGF-1 which maintained the cell phenotype and stimulated an anabolic response. However, data for IGF-1 treatments taken at time points later than day 4 (days 7, 11 and 14) did not demonstrate any significant differences in matrix deposition or release when compared with controls (data not shown). This suggests that the initial burst release of IGF-1has an effect on the cells but continued treatment at therapeutic levels does not, possibly due to the cells becoming refractory to continual exposure to the IGF-1. Despite this lack of effect at later time points, we believe that the increased levels of matrix production seen at the early time point suggests that binding IGF-1 to the scaffold may be beneficial by causing a more rapid stimulation of the cells in order to initiate extracellular matrix deposition and cartilage repair.

## Conclusions

The release of IGF-1 from this system was dependent on the IGF-1 loading concentration used to adsorb IGF-1 onto the CG scaffolds. And the amount of IGF-1 released into the media was highest at day 4. Relatively low levels of IGF-1 measured in the media may be attributed to the proteolytic degradation of free IGF-1, the degradation of IGF-1 after binding to the IGFR1 or the role of the scaffold and IGFBPs to act as an IGF-1 reservoir. OA chondrocytes seeded within scaffolds containing adsorbed IGF-1 deposited decorin and type II collagen in a dose dependent manner. The highest type II collagen deposition in this study was achieved via loading the scaffold with 50 μg/ml IGF-1. Cells seeded within the IGF-1 loaded scaffolds also deposited more ECM than the control group proving that the IGF-1 released from the scaffold remained bioactive and exerted an anabolic effect. The effectiveness of adsorbing IGF-1 onto the scaffold may be due to protection of the molecule from proteolytic digestion allowing a more sustained release of IGF-1 over time compared to adding multiple doses of exogenous IGF-1. Incorporating IGF-1 into the CG scaffold provided an initial therapeutic burst release of IGF-1 which is beneficial in initiating ECM deposition and repair in this in vitro model and shows potential for developing this delivery device in vivo.
